# Annotated type catalogue of *Bothriembryon* (Mollusca, Gastropoda, Orthalicoidea) in Australian museums, with a compilation of types in other museums

**DOI:** 10.3897/zookeys.194.2721

**Published:** 2012-05-17

**Authors:** Abraham S.H. Breure, Corey S. Whisson

**Affiliations:** 1 Netherlands Centre for Biodiversity Naturalis, P.O. Box 9517, Leiden, the Netherlands; 2 Western Australian Museum, Locked Bag 49, Welshpool, WA 6106

**Keywords:** Australia, Bothriembryontidae, types

## Abstract

Type material of 41 Australian *Bothriembryon* taxa present in Australian museums is critically listed, indicating systematic issues that need to be resolved in further studies. Information on additional type material of 22 taxa in non-Australian museums is compiled. The seven fossil taxa known so far are included in this catalogue. Based on the current systematic position, 38 species are treated in this paper. *Bothriembryon jacksoni* Iredale, 1939, *Bothriembryon notatus* Iredale, 1939, *Bothriembryon praecelsus* Iredale, 1939 and *Bothriembryon serpentinus* Iredale, 1939 are elevated to species level. *Bothriembryon gratwicki* (Cox, 1899) is listed as status to be determined.

## Introduction

The land snail genus *Bothriembryon* Pilsbry, 1894, is endemic to Australia but forms part of the Gondwanan element in the superfamily Orthalicoidea. This genus has received little in-depth attention, the last review being B.J. Smith (1992) summarizing all known data. Given the fact that *Bothriembryon* species are mostly patchy in their distribution, and in some areas species complexes have been identified, a revision of the genus is needed using modern techniques (Breure and Whisson, in preparation). An inventory of as much type material as possible would facilitate revisionary work; data of primary types for 63 taxa are presented herein. The aim of this paper is (1) to present additional data on the types of *Bothriembryon* in Australian museums (see also Kendrick and Wilson 1975, Wells 1977, B.J. Smith 1992); (2) to summarize recently published data for some European museums (Neubert and Janssen 2004, Köhler 2007, Breure and Ablett 2012), and add additional data from American and European museums; (3) to indicate systematic issues for several species to be clarified by further studies.

## Methods

For each taxon the original publication—in which the taxon was proposed—is mentioned, as well as papers in which reference is made to the type material. The type locality is quoted from the original publication in the original wording and language, with clarifying notes between square brackets. These localities have been mapped using SimpleMappr (Shorthouse 2010). The text of the original, or oldest, label is quoted, together with information from subsequent labels if containing information necessary for a correct interpretation. It should be noted that Iredale is considered to be somewhat remiss about marking his type material and that the designations of Iredale’s material in the Sydney museum has been done at a later stage by McMichael, presumably together with Iredale (A. Miller, pers. commun.). As a consequence this material has incorrectly been considered as holo- or paratypes by later authors (Wells 1975, B.J. Smith 1992). The original dimensions are quoted, as well as the dimensions of the type specimens; these have been taken with a digital calliper, using the methods figured by Breure (1974: fig. 2) for shell dimensions and Kendrick and Wilson (1975: fig. 1) for whorl counts; measurements up to 10 mm have an accuracy of 0.1 mm, those above 10 mm are accurate to 0.5 mm. Comparing the current measurements to those quoted from the original publication, one should be aware that the diameter especially may have been measured differently. In the case of syntypes, only the largest specimen has been measured. The number of specimens originally available, if quoted by the original author, is mentioned under remarks. Remarks are further given to describe any individual characteristics of the type specimens or any other details of the type lot. The current systematic position is given, following Breure and Romero (in press) for the classification at the family level, and B.J. Smith (1992) for the species level unless otherwise stated.

**Figure 1.**
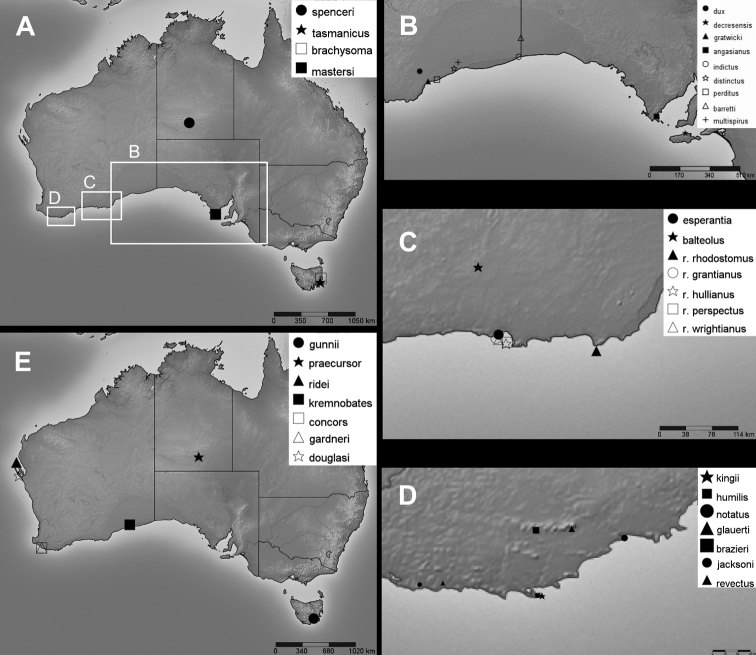
Type localities of *Bothriembryon* taxa, dealt with in this study, for which accurate type locality data are available. **A** Species of central and south-east Australia **B** Species from the eastern South Coast **C** Species from Esperance region **D** Species from Stirling Range and Albany regions **E** Fossil species.

**Figure 2.**
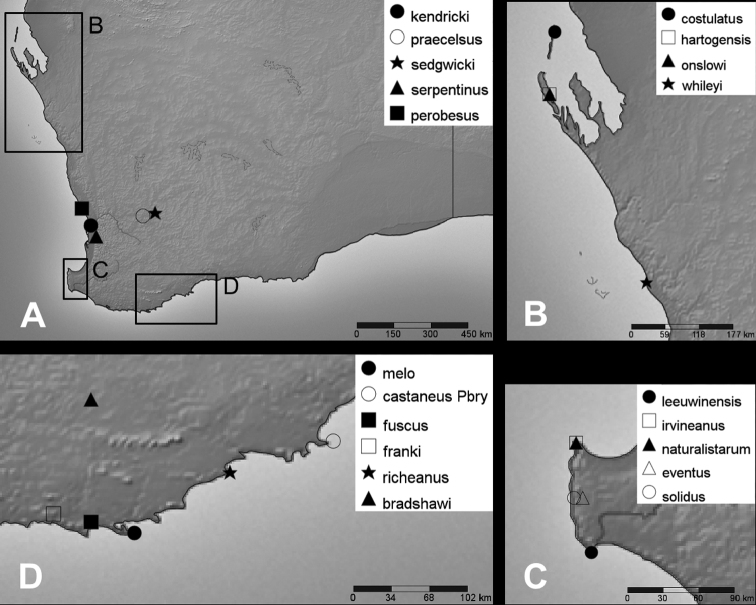
Type localities of *Bothriembryon* taxa (continued). **A** *Bothriembryon* species in Western Australia **B** Species of Shark Bay and adjacent region **C** Species of the Cape Leeuwin and Cape Naturaliste region **D** Species of Albany region.

Abbreviations used for depositories of material are: AM, Australian Museum, Sydney, Australia; ANSP, Academy of Natural Sciences, Philadelphia, USA; CPC, Geoscience Australia, Canberra, Australia; MHNG, Muséum d’Histoire Naturelle, Genève, Switzerland; MNHN, Muséum National d’Histoire Naturelle, Paris, France; MV, Museum of Victoria, Melbourne, Australia; NHMUK, Natural History Museum, London, U.K.; SAMA, South Australian Museum, Adelaide, Australia; SMF, Senckenberg Natur-Museum, Frankfurt am Main, Germany; TWCMS, Great Northern Museum (formerly Hancock Museum), Newcastle-upon-Tyne, U.K.; UMZC, University Museum of Zoology, Cambridge, U.K.; WAM, Western Australian Museum, Perth, Australia; ZMB, Museum für Naturkunde, Humboldt-Universität, Berlin, Germany. Other abbreviations used are: D, shell diameter; H, shell height; W, number of whorls. This paper follows the structure of Breure (2011) and Breure and Ablett (2012), with an additional section on types in non-Australian museums; given the recent papers by e.g. Neubert and Janssen (2004), Köhler (2007), and Breure and Ablett (2012), this sections presents only a compilation of information. Figs 1–2 presents the type localities of species. The dimensions of the specimens figured are presented in the text.

## Systematics

**Systematic list of nominal taxa of *Bothriembryon* in Australian museums**

Fossil taxa are indicated with an asterisk (*).

### Family Bothriembryontidae Iredale, 1939

**Remarks.** This genus was hitherto classified with the Bulimulidae (Pilsbry 1900, Breure 1979, B.J. Smith 1992). Recent phylogenetic studies of the superfamily show that the Gondwanan group within the Orthalicoidea (Placostylidae sensu Neubert et al. 2009, *Bothriembryon*, *Prestonella*, *Plectostylus*, and *Discoleus*) appears to be monophyletic (Breure et al. 2010, Breure and Romero in press). Thus the oldest family name, i.e. Bothriembryontidae, is used herein for this group.

***Bothriembryon*** Pilsbry, 1894

*Bothriembryon balteolus* Iredale, 1939; *Bothriembryon barretti* Iredale, 1930; *Bothriembryon bradshawi* Iredale, 1939; *Bothriembryon consors Kendrick, 1978; *Bothriembryon distinctus* Iredale, 1939; *Bothriembryon douglasi Kendrick, 1978; *Bothriembryon esperantia* Iredale, 1939; *Bothriembryon eventus* Iredale, 1939; *Bothriembryon franki* Iredale, 1939; *Bothriembryon gardneri Kendrick, 1978; *Bothriembryon glauerti* Iredale, 1939; *Bothriembryon grantianus* Iredale, 1939; *Bothriembryon gratwicki* (Cox, 1899); *Bothriembryon hullianus* Iredale, 1939; *Bothriembryon humilis* Pilsbry, 1900; *Bothriembryon indictus* Iredale, 1939; *Bothriembryon irvineanus* Iredale, 1939; *Bothriembryon jacksoni* Iredale, 1939; *Bothriembryon kendricki* Hill, Johnson & Merrifield, 1983; *Bothriembryon kremnobates Kendrick, 2005; *Bothriembryon notatus* Iredale, 1939; *Bothriembryon perditus* Iredale, 1939; *Bothriembryon perobesus* Iredale, 1939; *Bothriembryon perspectus* Iredale, 1939; *Bothriembryon praecelsus* Iredale, 1939; *Bothriembryon praecursor McMichael, 1968; *Bothriembryon revectus* Iredale, 1939; *Bothriembryon richeanus* Iredale, 1939; *Bothriembryon ridei Kendrick, 1978; *Bothriembryon sedgwicki* Iredale, 1939; *Bothriembryon serpentinus* Iredale, 1939; *Bothriembryon solidus* Pilsbry, 1900; *Bothriembryon whitleyi* Iredale, 1939; *Bothriembryon wrightianus* Iredale, 1939.

### Alphabetic list of taxa by species name

#### Bothriembryon balteolus

Iredale, 1939

http://species-id.net/wiki/Bothriembryon_balteolus

Fig. 6A

Bothriembryon balteolus Iredale 1939: 21, pl. 2 fig. 9; Wells 1977: 52; B.J. Smith 1992: 101.

##### Type locality.

[Western Australia] “Esperance Mallee Belt district, 50 miles south of Norseman, Madura, Salmon Gums”.

##### Label.

“Mallee belt / Esperance / WA”.

##### Dimensions.

“length 21 mm, breadth 15 mm”; lectotype H 21.5, D 13.6, W 5.0.

##### Type material.

WAM S15154, lectotype; AM C100716, paralectotype; AM C127557, three paralectotypes; AM C127558, two paralectotypes; WAM S15151, nine paralectotypes; WAM S15152, two paralectotypes; WAM S15153, two paralectotypes.

##### Remarks.

Wells (1977: 52) designated specimen WAM 9876 (now WAM S15154) as lectotype. Iredale’s description was based on “Many shells...”.

##### Current systematic position.

Bothriembryontidae, *Bothriembryon balteolus* Iredale, 1939.

#### Bothriembryon barretti

Iredale, 1930

http://species-id.net/wiki/Bothriembryon_barretti

Fig. 7A

Bothriembryon barretti Iredale 1930: 119, fig.; Iredale 1939: 35, pl. 2 figs 41–42; B.J. Smith 1992: 101.

##### Type locality.

[South Australia] “Nullabor Plain”.

##### Label.

“Nullabor Plains” “near Hampton / WA / fide Iredale 1939”.

##### Dimensions.

“length, 27 mm; breadth, 15.5 mm”; figured specimen H 28.1, D 16.5, W 5.3.

##### Type material.

AM C56628, two syntypes, C. Barrett leg.

##### Remarks.

The additional label information “near Hampton” (see also Iredale 1939: 36) probably refers to Hampton Tableland; this extends along the Eyre Highway between Eyre and Eucla.

##### Current systematic position.

Bothriembryontidae, *Bothriembryon barretti* Iredale, 1930.

#### Bothriembryon bradshawi

Iredale, 1939

http://species-id.net/wiki/Bothriembryon_bradshawi

Fig. 5F

Bothriembryon bradshawi Iredale 1939: 24, pl. 2 fig. 14; Wells 1977: 52; B.J. Smith 1992: 102.

##### Type locality.

[Western Australia] “Tambellup”.

##### Label.

“Tambellup / WA”.

##### Dimensions.

“Length, 19.5 mm., breadth, 12 mm”; figured specimen H 19.0, D 11.4, W 4.7.

##### Type material.

AM C100749, syntype; AM C59096, nine syntypes; AM C59208, 30+ syntypes; WAM S15148, four syntypes; WAM S15149, two syntypes; WAM S15150, one syntype (all ex E.R. Bradshaw).

##### Remarks.

Iredale’s description is based on “A good series” of shells. According to Wells (1977) the WAM collection holds five paratypes.

##### Current systematic position.

Bothriembryontidae, *Bothriembryon bradshawi* Iredale, 1939.

#### Bothriembryon inflatus castaneus

Pilsbry, 1900

http://species-id.net/wiki/Bothriembryon_inflatus_castaneus

Fig. 4C

Bothriembryon inflatus castaneus ‘Deshayes’ Pilsbry 1900: 5, pl. 1 fig. 18 [not fig. 11]; Kendrick and Wilson 1975: 315, pl. 1 figs 3a–3b; B.J. Smith 1992: 105.
Bothriembryon castaneus Pilsbry; Iredale 1939: 19, pl. 2 fig. 2.

##### Type locality.

[Western Australia] “Recherche Archipelago (*Dr. Cox*)”. See Remarks.

##### Label.

“Reserch [sic] / Archipelago / W. Australia”.

##### Dimensions.

“Alt. 22, diam. 13 (...) mill.”; figured specimen H 23.1, D 13.8, W 5.1.

##### Type material.

AM C87458, syntype (Cox coll.).

##### Remarks.

Kendrick and Wilson (1975), in their valuable study on type specimens of *Bothriembryon*, have argued why the type locality as given by Pilsbry is unlikely; they presume that the specimen originated from Doubtful Island in the Bremer Bay area. It should be noted that this locality is ca. 170 km east from Albany, Bald Head, which is the type locality of *Helix melo* Quoy and Gaimard, 1832, and of their variety *Helix melo castanea* (see next section). Should material from these two localities prove to be sufficiently distinct for systematic separation, it is worth noting that the name *castaneus* Pilsbry, 1900 is a homonym of *castanea* Quoy and Gaimard, 1832. The material is accompanied by a label “selected as type / by Iredale 1939 / p. 19”, but this is not regarded as a valid lectotype designation by Iredale.

##### Current systematic position.

Bothriembryontidae, *Bothriembryon melo* (Quoy and Gaimard, 1832).

#### Bothriembryon consors

Kendrick, 1978

http://species-id.net/wiki/Bothriembryon_consors

Figs 3C–E

Bothriembryon consors Kendrick 1978: 54, fig 4F–H.

##### Type locality.

“Windy Harbour, Western Australia. Shallow quarry NE of lighthouse beside track to Salmon Bay. Lat. 34°49'14"S, long. 116°00'52"E. Probably Pleistocene age. See Remarks.

##### Label.

“Windy Harbour, W.A. Shallow / quarry NE of lighthouse beside track / to Salmon Beach”, in Kendrick’s handwriting.

##### Dimensions .

“height of 29.9 mm, maximum diameter 13.8 mm”; holotype H 30.0, D 13.6, W 5.3.

##### Type material.

WAM 72421a, holotype; WAM 72421b–e, j and k, 70901a–b, 70160d, nine paratypes.

##### Remarks.

Although the formulation differs, this is the same locality where *Bothriembryon gardneri* Kendrick, 1978 has been found.

##### Current systematic position.

Bothriembryontidae, *Bothriembryon consors* Kendrick, 1978.

#### Bothriembryon decresensis

Cotton, 1940

http://species-id.net/wiki/Bothriembryon_decresensis

Fig. 5B

Bothriembryon decresensis Cotton 1940: 40; B.J. Smith 1992: 105.

##### Type locality.

[South Australia] “Cape Cassini, Kangaroo Island”.

##### Label.

“Cape Cassini / K.I.”

##### Dimensions.

“height 16 mm., width 10 mm”; holotype H 15.5, D 10.37, W 4.7 .

##### Type material.

SAMA D13773, holotype; SAMA D15588, 26 paratypes.

##### Remarks.

We tentatively follow the opinion of B.J. Smith (1992) that this taxon is synonymous with *Bothriembryon mastersi* (Cox, 1867). However, a more detailed study should corroborate this view.

##### Current systematic position.

Bothriembryontidae, *Bothriembryon mastersi* (Cox, 1867).

#### Bothriembryon distinctus

Iredale, 1939

http://species-id.net/wiki/Bothriembryon_distinctus

Fig. 7C

Bothriembryon distinctus Iredale 1939: 36, pl. 2 fig. 43; Wells 1977: 53; B.J. Smith 1992: 101.

##### Type locality.

[Western Australia] “Cardanumbi, west of Eyre”.

##### Label.

“Cardanumbi / WA”.

##### Dimensions.

“length of the type 27 mm., breadth 15.5 mm”; figured specimen H 26.3, D 14.5, W 6.5.

##### Type material.

AM C100727, syntype; AM C127568, three syntypes; WAM S15147, seven syntypes.

##### Remarks.

Iredale (1939) based himself on “A series of shells”. B.J. Smith (1992) synonimized this taxon with *Bothriembryon barretti* Iredale, 1930 after comparison of type specimens.

##### Current systematic position.

Bothriembryontidae, *Bothriembryon barretti* Iredale, 1930.

#### Bothriembryon douglasi

Kendrick, 1978

http://species-id.net/wiki/Bothriembryon_douglasi

Figs 3G–H

Bothriembryon douglasi Kendrick 1978: 55, fig. 6A–E.

##### Type locality.

“Sea cliff at the Carrarang-Tamala boundary fence, Edel Land, Shark Bay, Western Australia. Lat. 26°32'26"S, long. 113°26'42"E.

##### Label.

“Sea cliff at the Carrarang- / Tamala boundary fence, Edel Land / from top 25 feet / (7.5 m) of the cliff”, in Kendrick’s handwriting.

##### Dimensions.

“height of 34.2 mm, maximum diameter 19.6 mm”; holotype H 34.2, D 19.1, W 5.7.

##### Type material.

WAM 661036a, holotype; 661036b–c, 681434c, d, g, j, and o, seven paratypes. All material B.R. Wilson and G.W. Kendrick leg., 4.iii.1966.

##### Current systematic position.

Bothriembryontidae, *Bothriembryon douglasi* Kendrick, 1978.

#### Bothriembryon esperantia

Iredale, 1939

http://species-id.net/wiki/Bothriembryon_esperantia

Fig. 6B

Bothriembryon esperantia Iredale 1939: 21, pl. 2 fig. 8; Wells 1977: 53; B.J. Smith 1992: 103.

##### Type locality.

[Western Australia] “Esperance”.

##### Label.

“Esperance / WA”.

##### Dimensions.

“Length 23 mm., breadth 15 mm”; figured specimen H 23.9, D 15.0, W 5.2.

##### Type material.

AM C48681, 40+ syntypes; AM C100731, syntype; MV F333, 36 syntypes; MV F26930, four syntypes; WAM S15146, four syntypes.

##### Remarks.

Iredale gives two sets of measurements (“The largest measures 26 mm. by 16 mm.”), but does not mention the total number of specimens he examined.

##### Current systematic position.

Bothriembryontidae, *Bothriembryon esperantia* Iredale, 1939.

#### Bothriembryon leeuwinensis eventus

Iredale, 1939

http://species-id.net/wiki/Bothriembryon_leeuwinensis_eventus

Fig. 7H

Bothriembryon leeuwinensis eventus Iredale, 1939: 25, pl. 2 fig. 18; Wells 1977: 53; B.J. Smith 1992: 105.

##### Type locality.

[Western Australia] “Margaret River”.

##### Label.

“Margaret River / SWA”.

##### Dimensions.

“the type measuring 23 mm. in length by 14 mm. in breadth”; figured specimen H 22.0, D 12.2, W 4.9.

##### Type material.

AM C100771, syntype; AM C127657, three syntypes; WAM S15124, 11 syntypes.

##### Remarks.

Iredale (1939) refers to “Another series from Margaret River”; this may refer to the village “Margaret River”, or to a location along the Margaret River watercourse. In the latter case it is more likely to be in the coastal area.

##### Current systematic position.

Bothriembryontidae, *Bothriembryon leeuwinensis* (E.A. Smith, 1894).

#### Bothriembryon franki

Iredale, 1939

http://species-id.net/wiki/Bothriembryon_franki

Fig. 8B

Bothriembryon franki Iredale 1939: 29, pl. 2 fig. 25.
Bothriembryon fuscus franki Iredale; Smith 1992: 103.

##### Type locality.

[Western Australia] “Denmark”.

##### Label.

“Denmark / Deep River / WA”.

##### Dimensions.

“Length 40 mm., breadth 21 mm.”; figured specimen H 40.0, D 19.6, W 5.2.

##### Type material.

AM C100728, syntype; AM C127672, 35 syntypes; MV F3332, nine syntypes; MV F26911, four syntypes; WAM S15145, six syntypes.

##### Remarks.

The text in Iredale (1939) has led to some confusion with later authors. Under the heading “*Bothriembryon fuscus* Thiele 1930” Iredale described shells (“a very large series”) from Denmark as *Bothriembryon franki* (see the legend of his pl. 2; not *Bothriembryon fuscus franki* as Smith (1992) suggested). The type locality Deep River is ca. 100 km W Denmark in the Walpole area. The shells he referred to as collected by S.W. Jackson at “Deep River, Nornalup Inlet” were suggested to be identical with *Bothriembryon fuscus*, which was described by Thiele from Torbay. Wells (1977) erroneously listed the Deep River specimens as types of *fuscus* Thiele. Smith (1992) listed the same specimens as syntypes of *fuscus* from Torbay. See also *Bothriembryon fuscus* on p. 65.

##### Current systematic position.

Bothriembryontidae, *Bothriembryon fuscus* Thiele, 1930.

#### Bothriembryon gardneri

Kendrick, 1978

http://species-id.net/wiki/Bothriembryon_gardneri

Figs 3A–B, F

Bothriembryon gardneri Kendrick 1978: 51, fig. 4A–E.

##### Type locality.

“Point d’Entrecasteaux, Western Australia. Shallow quarry on crest of low ridge of calcarenite on north side of track from Windy Harbour to Salmon Beach. Lat. 34°49'14"S, long. 116°00'52" E“. Probably of Pleistocene age. See Remarks.

##### Label.

“Pt d’Entrecasteaux, WA. / Shallow quarry on low ridge, N side of / track from Windy Harbour to Salmon Beach”, in Kendrick’s handwriting.

##### Dimensions.

“height of 43.5 mm, maximum diamter 19.5 mm”; holotype H 43.5, D 18.4, W 5.8

##### Type material.

WAM 701603a, syntype; 701603b–c, 66794a–b, 66798a, h and w, seven syntypes; FMNH 194694/3, three paratypes. All material G. Gardner, J.K. Penglase, G.W. Kendrick leg, 1.viii.1970.

##### Remarks.

Although the formulation differs, this is the same locality where *Bothriembryon consors* Kendrick, 1978 has been found.

##### Current systematic position.

Bothriembryontidae, *Bothriembryon gardneri* Kendrick, 1978

#### Bothriembryon glauerti

Iredale, 1939

http://species-id.net/wiki/Bothriembryon_glauerti

Fig. 8C

Bothriembryon glauerti Iredale 1939: 29, pl. 2 fig. 24; Wells 1977: 53; B.J. Smith 1992: 103.

##### Type locality.

[Western Australia] “Stirling Ranges”.

##### Label.

“Stirling Range” (see Remarks).

##### Dimensions.

“The type measures 38 mm. in length by 22 mm. in breadth”; figured specimen H 36.7, D 20.8, W 5.5.

##### Type material.

WAM S14466, syntype; WAM S15144, three syntypes, F.R. Bradshaw leg.

##### Remarks.

According to the old WAM registration book (No. 10127) this material was collected by F.R. Bradshaw at Bluff Knoll. This more specific locality was not mentioned by Iredale (1939); the type locality is now restricted to Bluff Knoll. Iredale did not mention on how many specimens his description was based. The relationships between this taxon and *Bothriembryon fuscus* Thiele, 1930, *Bothriembryon franki* Iredale, 1939 and *Bothriembryon indutus* (Menke, 1843) need further study.

##### Current systematic position.

Bothriembryontidae, *Bothriembryon glauerti* Iredale, 1939.

#### Bothriembryon rhodostomus grantianus

Iredale, 1939

http://species-id.net/wiki/Bothriembryon_rhodostomus_grantianus

Fig. 6E

Bothriembryon rhodostomus grantianus Iredale 1939: 21, pl. 2 fig. 5; Wells 1977: 54; B.J. Smith 1992: 107.

##### Type locality.

[Western Australia, Recherche Archipelago] “Charley Island”.

##### Label.

“Charley I / Recherche Arch’o / WA”, “?Type / (?measurements) / Fig’d Spem”.

##### Dimensions.

“length, 40 mm. by breadth, 20 mm.”; figured specimen H 36.5, D 21.2, W 5.5.

##### Type material.

AM C100721, syntype; WAM S15118, four syntypes, A.F. Basset Hull leg., 1921.

##### Remarks.

The AM label indicates that this is the specimen figured by Iredale, but shows doubt about the status of the specimen. Iredale (1939) gives several sets of dimensions, but the ones quoted above are for the “type”; see introduction why we regard Iredale’s specimens generally as syntypes. The possible subspecific status of this taxon needs further confirmation.

##### Current systematic position.

Bothriembryontidae, *Bothriembryon rhodostomus* (Gray, 1834).

#### Bulimus (Liparus) gratwicki

Cox, 1899

http://species-id.net/wiki/Bulimus_gratwicki

Fig. 8D

Bulimus (Liparus) gratwicki Cox 1899: 435, figs 1–3; Smith 1992: 103.

##### Type locality.

“About 50 miles east of Israelite Bay, Western Australia, two miles from the edge of the cliffs”.

##### Label.

“50 miles E of / Israelite Bay / WA”, taxon label in Cox’ handwriting. See Remarks.

##### Dimensions.

“Length 30 mm. Width about the middle of the body whorl 10 mm”; holotype H 29.5, D 10.3, W 7.4.

##### Type material.

AM C6376, two syntypes; AM C127559, eight syntypes (ex Cox coll.).

##### Remarks.

According to B.J. Smith (1992) the two specimens in lot C6376 are syntypes; they are accompanied by a type written label stating “Type figd P.L.S.N.S.W. XXIV, p. 435 / Isrealite [sic] Bay, W.A. / Pres. Dr. J. C. Cox”. These specimens are, however, smaller than Cox stated and both are unlikely to have been figured. The largest specimen of lot C127599 fits the dimensions given by Cox better and matches the figure. These specimens are accompanied by the label mentioned above with Cox’ handwriting; a second label read reads “fr: near Israelite Bay / W.A.” and also bears the taxon name in the handwriting of Cox. The status of this taxon needs further clarification. The type locality is well known for fossil marine deposits; all museum material was collected as shells only, and the shape of this taxon is aberrant within the genus *Bothriembryon*, leaving doubt about its systematic classification.

##### Current systematic position.

Status to be determined.

#### Bothriembryon rhodostomus hullianus

Iredale, 1939

http://species-id.net/wiki/Bothriembryon_rhodostomus_hullianus

Fig. 6G

Bothriembryon rhodostomus hullianus Iredale 1939: 20, pl. 2 fig. 4; Wells 1977: 54; B.J. Smith 1992: 106.

##### Type locality.

[Western Australia, Recherche Archipelago] “Gunton Island”.

##### Label.

“Gunton I / Recherche Arch’o / WA”.

##### Dimensions.

“35 mm. in length by 18 mm. in breadth”; figured specimen H 31.7, D 18.3, W 6.0.

##### Type material.

AM C100718, syntype; WAM S15116, four syntypes, A.F. Basset Hull leg., 1921.

##### Remarks.

The AM specimen is labelled “holotype” (not in Iredale’s but in McMichael’s hand), but does not confirm with the original measurements; moreover, Iredale (1939) states he had more specimens (“shells”). Therefore we consider it a syntype. The possible subspecific status of this taxon needs further confirmation.

##### Current systematic position.

Bothriembryontidae, *Bothriembryon rhodostomus* (Gray, 1834).

#### Bothriembryon physoides humilis

Pilsbry, 1900

http://species-id.net/wiki/Bothriembryon_physoides_humilis

Fig. 9B

Bothriembryon physoides humilis Pilsbry 1900: 10, pl. 2 figs 33–34; B.J. Smith 1992: 104.
Bothriembryon kingii (Gray); Iredale 1939: 31, pl. 2 fig. 28.

##### Type locality.

“Western Australia, King George Sound”.

##### Label.

“King George Sound”.

##### Dimensions.

“Alt. 17.5, diam. 10 (...) mill.”; lectotype H 17.2, D 10.1, W 4.8.

##### Type material.

AM C100774, lectotype; AM C1062, two paralectotypes; ANSP 65568, two paralectotypes (dry) and three bodies (alcohol).

##### Remarks.

In his original description, Pilsbry (1900) listed the dimensions of two shells, which originated from Hedley resp. Cox. The latter specimen was apparently regarded by Iredale (1939) as “the type of Pilsbry’s var. *humilis*”. This is to be regarded as a lectotype designation (ICZN Art. 74.6); Smith (1992) erroneously regarded it as holotype. Later, Baker (1963: 228) erroneously selected the shell figured in Pilsbry 1900 pl. 2 fig. 34 as lectotype (ANSP 65568a).

**Current systematic position.** Bothriembryontidae, *Bothriembryon kingii* (Gray, 1825).

#### Bothriembryon barretti indictus

Iredale, 1939

http://species-id.net/wiki/Bothriembryon_barretti_indictus

Fig. 7B

Bothriembryon barretti indictus Iredale 1939: 36; B.J. Smith 1992: 101.

##### Type locality.

[Western Australia] “Eucla”.

##### Label.

“Eucla / SWA”.

##### Dimensions.

“30 mm. in length by 15 mm. in width”; figured specimen H 28.8, D 15.6, W 5.7.

##### Type material.

AM C100730, syntype; AM C127539, three syntypes; AM C532, three syntypes.

##### Remarks.

Iredale (1939) states “The narrow form was figured by Pilsbry [1900], f. 63, and his specimen is here refigured as type of a subspecies, *Bothriembryon barretti indictus* nov.”. Since Iredale mentioned to have had more shells available, we consider all material as syntypes.

##### Current systematic position.

Bothriembryontidae, *Bothriembryon barretti* Iredale, 1930.

#### Bothriembryon irvineanus

Iredale, 1939

http://species-id.net/wiki/Bothriembryon_irvineanus

Fig. 7F

Bothriembryon irvineanus Iredale 1939: 24, pl. 2 fig. 15; Wells 1977: 53 (as *iruineanus* [sic]); B.J. Smith 1992: 104.

##### Type locality.

[Western Australia] “Cape Naturaliste”.

##### Label.

“Cape Naturaliste / SWA”.

##### Dimensions.

“Length of type, 26 mm.: breadth, 16 mm.”; figured specimen H 24.2, D 19.6, W 5.1.

##### Type material.

AM C100773, syntype; AM C127707, nine syntypes; WAM S15143, three syntypes, Mrs. Irvine leg., 1892.

##### Remarks.

Iredale (1939) based himself on “a series” of shells; we consider therefore all material as syntypes.

##### Current systematic position.

Bothriembryontidae, *Bothriembryon irvineanus* Iredale, 1939.

#### Bothriembryon jacksoni

Iredale, 1939

http://species-id.net/wiki/Bothriembryon_jacksoni

Fig. 9C

Bothriembryon jacksoni Iredale 1939: 31, pl. 2 fig. 30; Wells 1977: 53; B.J. Smith 1992: 105.

##### Type locality. 

[Western Australia] “Deep River, Frankland River, Nornalup”.

##### Label.

“Deep River / SWA”.

##### Dimensions.

“The length of the type is 27 mm., the breadth 15 mm.”; figured specimene H 27.2, D 14.2, W 5.4.

##### Type material.

AM C100725, syntype; WAM S15142, six syntypes, S.W. Jackson leg., xii.1912.

##### Remarks.

Iredale (1939) did not explicitly state on how many specimens his description was based. This taxon is part of the *Bothriembryon kingii* species complex that extends from the Walpole area in the west to near Hopetoun in the east. Smith (1992) placed this species in the synonymy of *Bothriembryon kingii* (Gray, 1825). However, we are of the opinion that this species groupneeds further study to ascertain the systematic position of this taxon.

##### Current systematic position.

Bothriembryontidae, *Bothriembryon jacksoni* Iredale, 1939.

#### Bothriembryon kendricki

Hill, Johnson & Merrifield, 1983

http://species-id.net/wiki/Bothriembryon_kendricki

Fig. 4F

Bothriembryon kendricki Hill et al. 1983: 238, figs 3–4; B.J. Smith 1992: 104.

##### Type locality.

[Western Australia] “King’s Park, Perth”.

##### Label.

“King’s Park, Perth”.

##### Dimensions.

“height 16.24 mm; width 11.86 mm”; holotype H 16.2, D 11.86, W 4.0.

##### Type material.

WAM S14552, holotype; WAM S4009, eight paratypes; WAM S15125, nine paratypes; WAM S15126, two paratypes; WAM S15127, seven paratypes; WAM S15128, one paratype; WAM S15129, four paratypes; WAM S15130, two paratypes; WAM S15131, five paratypes; WAM S15132, four paratypes; WAM S15133, one paratype; WAM S15134, four paratypes; WAM S15135, one paratype; WAM S15136, 18 paratypes; WAM S15137, four paratypes; WAM S15138, 12 paratypes; WAM S15139, seven paratypes; WAM S15140, one paratype; WAM S15141, one paratype; WAM S15526, four paratypes.

##### Remarks.

The paratypes are—if not from the type locality—from several localities in and around Perth; see Hill et al. 1983: 239.

##### Current systematic position.

Bothriembryontidae, *Bothriembryon kendricki* Hill, Johnson & Merrifield, 1983.

#### Bothriembryon kremnobates

Kendrick, 2005

http://species-id.net/wiki/Bothriembryon_kremnobates

Figs 3I–J

Bothriembryon kremnobates Kendrick 2005: 310, fig. 1A.

##### Type locality.

“Roe Plains, Madura district, Western Australia (...) Late Pliocene”.

##### Label.

“Roe Plains, Madura district, / W.A. Pit 0.5 km N of Hampton Microwave / Repeater Tower. Carbonate sand with PTO / large shells; 0.7–1.1 m / above base of formation / (Roe Calcarenite). Note matrix / within shell is same as Roe / Calcarenite”, in Kendrick’s handwriting.

##### Dimensions.

“Shell height 21.5, max. diameter 14.7 (mm)”; holotype H 21.5, D 14.7, W 5.4.

##### Type material.

WAM 81.847, holotype, V.A. Ryland and G.W. Kendrick leg., 29.ix–4.x.1980; WAM 81.1762, one paratype; WAM 81.1774, one paratype. Paratypes V.A. Ryland, G.W. and W.E. Kendrick leg., 20–23.ix.1976.

##### Current systematic position.

Bothriembryontidae, *Bothriembryon kremnobates* Kendrick, 2005.

#### Bulimus mastersi

Cox, 1867

http://species-id.net/wiki/Bulimus_mastersi

Fig. 5A

Bulimus mastersi Cox 1867: 39; B.J. Smith 1992: 105.

##### Type locality.

“Port Lincoln, South Australia (*Masters*)”.

##### Label.

“Flinders Is., S.A.”, in a later handwriting.

##### Dimensions.

“Long. 0.74, diam. 0.45 unc. [H 18.8, D 11.43 mm]”; figured specimen H 17.4, D 11.77, W 4.7.

##### Type material.

SAMA D11341, one possible syntype.

##### Remarks.

The specimen is slightly smaller than Cox’ original dimensions, and the label is different from that given by Cox (1867). It is considered a possible syntype as there remains some doubt if this shell was part of the original series.

##### Current systematic position.

Bothriembryontidae, *Bothriembryon mastersi* (Cox, 1867).

#### Bothriembryon multispirus

Macpherson, 1951

http://species-id.net/wiki/Bothriembryon_multispirus

Fig. 7E

Bothriembryon multispirus Macpherson 1951: 30; B.J. Smith 1992: 101.

##### Type locality.

[Western Australia] “20 miles west of Cocklebiddy Waterhole”.

##### Label.

“20 miles west of Cocklebiddy Water / Hole, W.A.”.

##### Dimensions.

“Length 24 mm.; breadth 12 mm.”; holotype H 24.2, D 12.7, W 6.7.

##### Type material.

MV F5716, holotype; F3068, F3073 four resp. two paratypes, Russell Grimwade expedition, ix.1947.

##### Remarks.

B.J. Smith (1992) placed this taxon in the synonymy of *Bothriembryon barretti* Iredale, 1930, based on examination of the types.

##### Current systematic position.

Bothriembryontidae, *Bothriembryon barretti* Iredale, 1930.

#### Bothriembryon notatus

Iredale, 1939

http://species-id.net/wiki/Bothriembryon_notatus

Fig. 9D

Bothriembryon notatus Iredale 1939: 31, pl. 2 fig. 29; Wells 1977: 54; B.J. Smith 1992:  104.

##### Type locality.

[Western Australia] “Pallinup River”.

##### Label.

“Pallenup R / SWA”.

##### Dimensions.

“Length 24 mm., breadth 11 mm.”; figured specimen H 23.6, D 10.9, W 5.2.

##### Type material.

AM C100726, syntype; AM C127661, four syntypes; WAM S15121, seven syntypes; WAM S15122, six syntypes; WAM S15123, six syntypes.

##### Remarks.

This taxon is part of the *Bothriembryon kingii* species complex that extends from the Walpole area in the west to near Hopetoun in the east. Smith (1992) placed this species in the synonymy of *Bothriembryon kingii* (Gray, 1825). However, we are of the opinion that this species groupneeds further study.

##### Current systematic position.

Bothriembryontidae, *Bothriembryon notatus* Iredale, 1939.

#### Bulimus onslowi

Cox, 1864

http://species-id.net/wiki/Bulimus_onslowi

Fig. 5H

Bulimus onslowi Cox 1864: 185; Kendrick and Wilson 1975: 308, pl. 5 figs 1a–b; B.J. Smith 1992: 106.

##### Type locality.

“Dirk Hartog’s Island, Shark Bay, Western Australia”.

##### Label.

See Remarks.

##### Dimensions.

“Long. 0.85, diam. 0.60 unc. [H 21.6, D 15.2 mm]”; figured specimen H 21.7, D 13.9, W 4.6.

##### Type material.

AM C84882, syntype.

##### Remarks.

Kendrick and Wilson (1975) have discussed the type specimen, clarifying that the original label has been lost and that “the dimensions differ slightly from those given by Cox”.

##### Current systematic position.

Bothriembryontidae, *Bothriembryon onslowi* (Cox, 1864).

#### Bulimus indutus pallidus

Tate, 1879

http://species-id.net/wiki/Bulimus_indutus_pallidus

Fig. 7D

Bulimus indutus pallidus Tate 1879: 135; B.J. Smith 1992: 101. Not *Bulimus pallidus* C.B. Adams, 1845.

##### Type locality.

[South Australia, Nullarbor Plain] “Bunda Plateau”.

##### Label.

“Nullarbor Plain”.

##### Dimensions.

Not given; figured specimen H 31.0, D 17.0, W 5.8.

##### Type material.

AM C477, three syntypes.

##### Remarks.

The specimens are labelled as “paratypes”; however, it is unclear on which evidence this is based and the specimens are herein considered as syntypes.

##### Current systematic position.

Bothriembryontidae, *Bothriembryon barretti* Iredale, 1930.

#### Bothriembryon perditus

Iredale, 1939

http://species-id.net/wiki/Bothriembryon_perditus

Fig. 7I

Bothriembryon perditus Iredale 1939: 32, pl. 2 fig. 32; Wells 1977: 54; B.J. Smith 1992: 106.

##### Type locality.

[Western Australia] “70 miles east of Israelite Bay”.

##### Label.

“70M E of / Israelite Bay / WA”.

##### Dimensions.

“Length of type 24 mm., breadth 12 mm.”; figured specimen H 23.2, D 11.6, W 5.3.

##### Type material.

AM C100720, syntype; AM C127567, three syntypes; WAM S15120, seven syntypes, E. Gatewick leg.

##### Remarks.

From his description it is clear that Iredale (1939) based himself on more than one specimen.

##### Current systematic position.

Bothriembryontidae, *Bothriembryon perditus* Iredale, 1939.

#### Bothriembryon perobesus

Iredale, 1939

http://species-id.net/wiki/Bothriembryon_perobesus

Fig. 9G

Bothriembryon perobesus Iredale 1939: 28, pl. 2 fig. 22; Wells 1977: 54; B.J. Smith 1992: 106.

##### Type locality.

[Western Australia] “the mouth of the Moore River”.

##### Label.

The material is accompanied by three labels. A very thin fragile old paper label with fine ink reads “Moore River, WA”; an old thicker card label with thicker black ink says “mouth of Moore River” and a lined paper label with pencil says “Moore River (mouth of)”.

##### Dimensions.

“The height of the shell is 25 mm., while its breadth is 19 mm.”; holotype H 24.2, D 17.9, W 4.9

##### Type material.

WAM S14467, holotype.

##### Remarks.

Iredale (1939) mentioned to have “One specimen” available; therefore we consider this as the holotype. The current taxonomic status of the species needs confirmation.

##### Current systematic position.

Bothriembryontidae, *Bothriembryon perobesus* Iredale, 1939.

#### Bothriembryon rhodostomus perspectus

Iredale, 1939

http://species-id.net/wiki/Bothriembryon_rhodostomus_perspectus

Fig. 6F

Bothriembryon rhodostomus perspectus Iredale 1939: 21, pl. 2 fig. 7; Wells 1977: 54; B.J. Smith 1992: 107.

##### Type locality.

[Western Australia, Recherche Archipelago] “Woody Isle”.

##### Label.

“Woody I / Recherche Arch’o / WA”.

##### Dimensions.

“length, 31 mm by 17 mm., and 30 mm. by 18 mm.”; figured specimen H 34.5, D 20.0, W 5.9.

##### Type material.

AM C100719, syntype; WAM S15117, two syntypes, A.F. Basset Hull leg., 1921.

##### Remarks.

Iredale (1939) gave two sets of measurements. The specimen in the AM collection does not confirm to these measurements, but bears an original label with the indication “type”; we consider it a syntype but not the holotype. The possible subspecific status of this taxon needs further confirmation.

##### Current systematic position.

Bothriembryontidae, *Bothriembryon rhodostomus* (Gray, 1834).

#### Bothriembryon praecelsus

Iredale, 1939

http://species-id.net/wiki/Bothriembryon_praecelsus

Fig. 5I

Bothriembryon praecelsus Iredale 1939: 22, pl. 2 fig. 11; Wells 1977: 54; B.J. Smith 1992: 102.

##### Type locality.

[Western Australia] “Kellerberrin”.

##### Label.

“Kellerberrin”.

##### Dimensions.

“length 29 mm., breadth 20 mm.”; figured specimen H 27.1, D 17.9, W 4.8

##### Type material.

WAM S14468, holotype.

##### Remarks.

Iredale (1939) mentioned to have “One specimen” available; therefore we consider this as the holotype. This species, which was synonymized by Smith (1992) with *Bothriembryon bulla* (Menke, 1843), is now thought to be extinct (DEC 2010).

##### Current systematic position.

Bothriembryontidae, *Bothriembryon praecelsus* Iredale, 1939.

#### Bothriembryon praecursor

McMichael, 1968

http://species-id.net/wiki/Bothriembryon_praecursor

Fig. 3M

Bothriembryon praecursor McMichael 1968: 149, pl. 11 figs 7–9; Crespin 1974: 125.

##### Type locality.

[Northern Territory] “4 miles east-north-east of Deep Well homestead, near Alice Springs, N.T.”.

##### Label.

No labels, see Remarks.

##### Dimensions.

“Maximum height 14.00, maximum diameter 9.00 (mm)”; holotype H 14.17, D 9.03, W 3+.

##### Type material.

CPC6906, holotype; CPC6907–CPC6909, three paratypes.

##### Remarks.

This is a fossil species from Tertiary age; no further details are known about the dating of the horizon where the shells were found. “The holotype is an isolated steinkern of an immature shell” (McMichael 1968); the top of the shell is damaged. The paratypes are larger, up to 20.0 mm. The specimens are not accompanied with labels, but Crespin (1974) provides as data “6.5 km ENE of Deep Well hstd [homestead], near Alice Springs, N.T. (loc. Rd21)” [Holotype and paratypes 1–2], “18 km NE of Deep Well hstd, near Alice Springs, N.T. (loc. NT409)” [Paratype 3].

##### Current systematic position.

Bothriembryontidae, *Bothriembryon praecursor* McMichael, 1968.

#### Bothriembryon revectus

Iredale, 1939

http://species-id.net/wiki/Bothriembryon_revectus

Fig. 9E

Bothriembryon revectus Iredale 1939: 33, pl. 2 fig. 37; B.J. Smith 1992: 106.

##### Type locality.

[Western Australia] “Bow River”.

##### Label.

“Bow River / SWA”.

##### Dimensions.

“23 mm. in length by 11 mm. in breadth”; figured specimen H 21.8, D 11.2, W 5.2.

##### Type material.

AM C100723, syntype; AM C127676, seven syntypes; AM C127678, 15 syntypes; AM C127677, two syntypes; WAM S15119, four syntypes, S.W. Jackson, 27.x.1912.

##### Remarks.

Iredale (1939) mentioned to have “some shells” available for his description.

##### Current systematic position.

Bothriembryontidae, *Bothriembryon revectus* Iredale, 1939.

#### Bothriembryon richeanus

Iredale, 1939

http://species-id.net/wiki/Bothriembryon_richeanus

Fig. 9F

Bothriembryon richeanus Iredale 1939: 24, pl. 2 fig. 16; B.J. Smith 1992: 107.

##### Type locality.

[Western Australia] “Cape Riche”.

##### Label.

“Cape Riche / SWA”.

##### Dimensions.

“24 mm. in length, 13 mm. in breadth”; figured specimen H 23.4, D 12.5, W 5.1.

##### Type material.

AM C100772, syntype; AM C127709, three syntypes; WAM S15109, one syntype (ex Brazier coll.).

##### Remarks.

Iredale (1939) based himself on “a series” of shells.

##### Current systematic position.

Bothriembryontidae, *Bothriembryon richeanus* Iredale, 1939.

#### Bothriembryon ridei

Kendrick, 1978

http://species-id.net/wiki/Bothriembryon_ridei

Figs 3K–L

Bothriembryon ridei Kendrick 1978: 56, fig. 6 F–H.

##### Type locality.

“Western side of Dorre Island, Western Australia; limestone cliffs opposite Disaster Cove. Lat. 24°59'52"S, long. 113°07'12"E. Probable Pleistocene age.”

##### Label.

“Dorre Is., W.A., West / side opposite Disaster Cove”, in Kendrick’s handwriting.

##### Dimensions.

“height of 36.3 mm, maximum diameter 22.7 mm”; holotype H 36.6, D 22.5, W 5.8.

##### Type material.

WAM 60.434a, holotype; 60.434b, d–e, 66.660a, 74.531a, five paratypes. W.D.L. Ride leg.

##### Current systematic position.

Bothriembryontidae, *Bothriembryon ridei* Kendrick, 1978.

#### Bothriembryon sedgwicki

Iredale, 1939

http://species-id.net/wiki/Bothriembryon_sedgwicki

Fig. 5G

Bothriembryon sedgwicki Iredale 1939: 22, pl. 2 fig. 12; B.J. Smith 1992: 107.

##### Type locality.

[Western Australia] “Nangeenan via Merredin”.

##### Label. 

“Nangeenan / WA”.

##### Dimensions.

“Height 17 mm., breadth 11 mm.”; figured specimen H 15.9, D 10.3, W 4.5.

##### Type material.

AM C100831, syntype; AM C127706, two syntypes; AM C127707, 11 syntypes; WAM S15108, seven syntypes, E. Sedgwick leg.

##### Remarks.

Iredale (1939) had “a series including juveniles” available for his description.

##### Current systematic position.

Bothriembryontidae, *Bothriembryon sedgwicki* Iredale, 1939.

#### Bothriembryon serpentinus

Iredale, 1939

http://species-id.net/wiki/Bothriembryon_serpentinu

Fig. 4G

Bothriembryon serpentinus Iredale 1939: 22, pl. 2 fig. 10; Wells 1977: 55; B.J. Smith 1992: 104.

##### Type locality.

[Western Australia] “Serpentine Falls, Darling Range”.

##### Label.

“Serpentine Falls / WA”.

##### Dimensions.

“28 mm. in length and 16 mm. in breadth”; figured specimen H 27.5, D 15.4, W 5.2.

##### Type material.

AM C100724, syntype; AM C127679, six syntypes; WAM S15107, 50 syntypes, Glauert leg., 1.vi.1927.

##### Remarks.

The original description was based on “A large series of shells”. Smith (1992) mentioned 60 paratypes in the WAM collection. He synonimized this taxon with *Bothriembryon indutus* (Menke, 1843). We consider *Bothriembryon serpentinus* a distinct taxon, but this needs confirmation by anatomical and molecular research.

##### Current systematic position.

Bothriembryontidae, *Bothriembryon serpentinus* Iredale, 1939.

#### Bothriembryon kingii solidus

Pilsbry, 1900

http://species-id.net/wiki/Bothriembryon_kingii_solidus

Fig. 9H

Bothriembryon kingii solidus Pilsbry 1900: 9, pl. 2 fig. 28; B.J. Smith 1992: 107.
Bothriembryon sayi (Pfeiffer); Iredale 1939: 33, pl. 2 fig. 34.

##### Type locality.

“Western Australia, exact habitat unknown”; restricted to Margaret River (Iredale 1939: 33).

##### Label.

“Western / Australia”.

##### Dimensions.

Not given. Figured specimen H 31.8, D 13.2, W 6.0.

##### Type material.

AM C100722, syntype; AM C127708, two syntypes (ex Cox coll.).

##### Remarks.

As it is clear that this material was seen by Pilsbry, this material is herein considered as syntypes. It is not clear whether Iredale referred to “Margaret River” as a place or as geographical feature. The latter Margaret River flows near Cape Mentelle into the Indian Ocean; Cape Freychinet—type locality of *Bothriembryon sayi* (Pfeiffer, 1847)—is ca. 20 km to the south.

##### Current systematic position.

Bothriembryontidae, *Bothriembryon sayi* (Pfeiffer,  1847).

#### Liparus spenceri

Tate, 1894

http://species-id.net/wiki/Liparus_spenceri

Fig. 7K

Liparus spenceri Tate 1894: 192; Tate 1896: 202, pl. 17 fig. 3; B.J. Smith 1992: 107.

##### Type locality.

[Northern Territory, near Alice Springs] “Glen of Palms by the junction with Palm Creek” (Tate 1896: 202).

##### Label.

“Glen of Palms C.A.”.

##### Dimensions.

“Length, 20; width 12.5 (...) mm”; holotype H 20.0, D 13.36, W 4.5.

##### Type material.

SAMA D3171, holotype; SAMA D11333, 12 paratypes; SAMA D15578, four paratypes.

##### Remarks.

This material was collected during the Horn Scientific Expedition to Central Australia in 1894. Further type material is present in the Natural History Museum in London (Breure and Ablett 2012).

##### Current systematic position.

Bothriembryontidae, *Bothriembryon spenceri* (Tate, 1894).

#### Bothriembryon whitleyi

Iredale, 1939

http://species-id.net/wiki/Bothriembryon_whitleyi

Fig. 7J

Bothriembryon whitleyi Iredale 1939: 27, pl. 2 fig. 21; B.J. Smith 1992: 107.

##### Type locality.

[Western Australia] “Geraldton”.

##### Label.

“Geraldton / WA”.

##### Dimensions.

“Height 16 mm., breadth 12.5 mm.”; figured specimen H 14.4, D 10.0, W 4.5.

##### Type material.

AM C100729, syntype; AM C127615, 31 syntypes; AM C127713, three syntypes; WAM S1368, six syntypes; WAM S15106, one syntype, G.P. Whitley leg.

##### Remarks.

**Figure 7.**
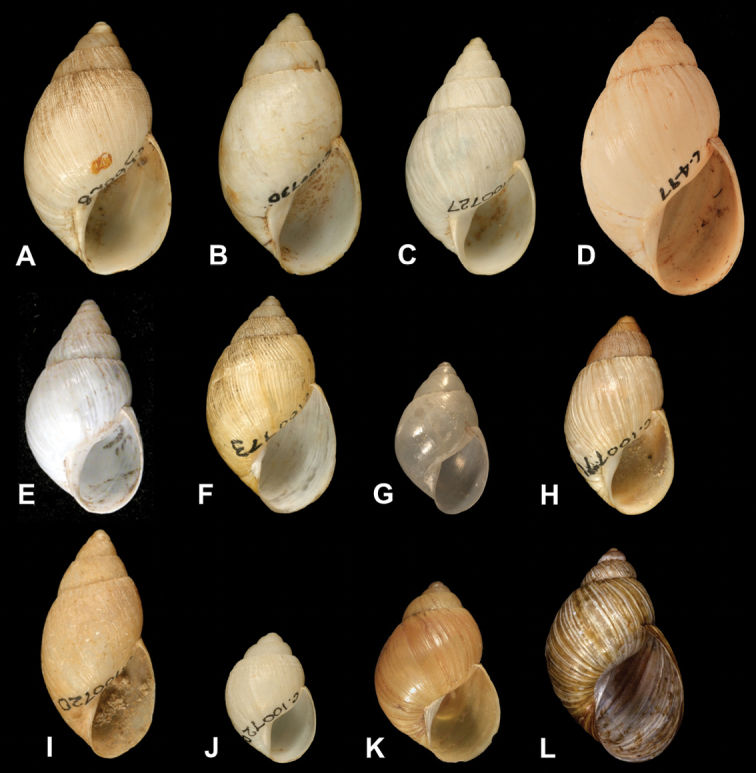
**A–E** *Bothriembryon barretti* Iredale, 1939 **A** Syntype, AM C56628 (H = 28.1) **B** Syntype of *B. indictus* Iredale, 1939, AM C100730 (H = 28.8) **C** Syntype of *B. distinctus* Iredale, 1939, AM C100727 (H = 26.3) **D** Syntype of *Bulimus industus pallidus* Tate, 1879, AM C477 (H = 31.0) **E** Holotype of *B. multispirus* Macpherson, 1951, MV F5716 (H = 24.2) **F** *B. irvineanus* Iredale, 1939, syntype, AM C100773 (H = 24.2) **G** *B. naturalistarum* Kobelt, 1901, syntype, SMF 25891/1a (H = 17.0) Photo: E. Neubert **H** *B. leeuwinensis* (E.A. Smith, 1894), syntype of *B. leeuwinensis eventus* Iredale, 1939, AM C100771 (H = 22.0) **I** *B. perditus* Iredale, 1939, syntype, AM C100720 (H = 23.2) **J** *B. whitleyi* Iredale, 1939, syntype, AM C100729 (H = 14.4) **K** *B. spenceri* (Tate, 1894), holotype, SAM D3171 (H = 20.0) **L** *B. tasmanicus* (Pfeiffer, 1853), holotype of *B. gunni brachysoma* Pilsbry, 1900, ANSP 8461 (H = 20.4) Photo: A. Lawless.

The shell in Fig. 7J is somewhat smaller than Iredale (1939) stated, but agrees with the specimen figured by him; the description is based on “A nice series”. This species is now thought to be extinct (DEC 2010).

##### Current systematic position.

Bothriembryontidae, *Bothriembryon whitleyi* Iredale, 1939.

#### Bothriembryon rhodostomus wrightianus

Iredale, 1939

http://species-id.net/wiki/Bothriembryon_rhodostomus_wrightianus

Fig. 6D

Bothriembryon rhodostomus wrightianus Iredale 1939: 21, pl. 2 fig. 6; Wells 1977: 54; B.J. Smith 1992: 107.

##### Type locality.

[Western Australia, Recherche Archipelago] “Rabbit Island”.

##### Label.

“Rabbit I. / Recherche Arch’o / WA”.

##### Dimensions.

“length 36 mm. by breadth 21.5 mm.”; figured specimen H 29.4, D 16.6, W 4+ (see Remarks).

##### Type material.

AM C100717, syntype; AM C48677, 19 syntypes; MV F26933, four syntypes; WAM S15110, five syntypes, A.F. Basset Hull leg., 1921.

##### Remarks.

The top of the specimen figured by Iredale (1939) is damaged; it may be noted that the measurements deviate from the published ones and the description was based on “a fine series”. The possible subspecific status of this taxon needs further confirmation.

##### Current systematic position.

Bothriembryontidae, *Bothriembryon rhodostomus* (Gray, 1834).

### Compilation of *Bothriembryon* type material in non-Australian museums

Additional to the data presented above, the following type specimens have been located in non-Australian museums. References to recently figured type specimens have been added; some other taxa are (re-)figured. Taxa are listed as originally published, arranged alphabetically on species name.

#### Bulimus angasianus

Pfeiffer, 1864

http://species-id.net/wiki/Bulimus_angasianus

Bulimus angasianus Pfeiffer 1864: 528; Breure and Ablett 2012: 5, figs 2A–B.

##### Type material.

NHMUK 1870.10.26.176, six syntypes; TWCMS T0230.1–3, three syntypes.

##### Current systematic position.

Bothriembryontidae, *Bothriembryon angasianus* (Pfeiffer, 1864).

#### Bulimus baconi

Benson, 1854

http://species-id.net/wiki/Bulimus_baconi

Fig. 4E

Bulimus baconi Benson 1854: 99.

##### Type material.

UMZC 2397, holotype.

##### Remarks.

We follow B.J. Smith (1992) for the systematic position of this taxon.

##### Current systematic position.

Bothriembryontidae, *Bothriembryon bulla* (Menke, 1843).

#### Bothriembryon gunni brachysoma

Pilsbry, 1900

http://species-id.net/wiki/Bothriembryon_gunni_brachysoma

Fig. 7L

Bothriembryon gunni brachysoma Pilsbry 1900: 19, pl. 3 fig. 53.

##### Type material.

ANSP 8461, holotype.

##### Remarks.

This taxon was placed in the synonymy of *Bothriembryon tasmanicus* (Pfeiffer, 1853) by Iredale (1937: 313). We tentatively follow his judgement, but further study should clarify the status of this taxon.

##### Current systematic position.

Bothriembryontidae, *Bothriembryon tasmanicus* (Pfeiffer, 1853).

#### Bulimus (Liparus) brazieri

Angas, 1871

http://species-id.net/wiki/Bulimus_brazieri

Bulimus (Liparus) brazieri Angas 1871: 19, pl. 1 fig. 28; Breure and Ablett 2012: 7, figs 2C–E, 2ii

##### Type material.

NHMUK 1870.11.5.8, lectotype, and one paralectotype (Angas coll.).

##### Current systematic position.

Bothriembryontidae, *Bothriembryon brazieri* (Angas, 1871).

#### Helix melo castanea

Quoy & Gaimard, 1832

http://species-id.net/wiki/Helix_melo_castanea

Fig. 4B

Helix melo castanea Quoy and Gaimard 1832: 109, pl. 9 figs 6–7; Kendrick and Wilson 1975: 315, pl. 1 figs 2a–2b.

##### Type material.

MNHN 24645 (see Remarks).

##### Remarks.

This taxon was described as “Varietas castanea; vitta alba cincta” by Quoy and Gaimard (1832); together with their figures we consider this an indication according to Art. 11.4.3. ICZN. This taxon has been ascribed incorrectly to Deshayes (1838: 245), who merely copied the text and figures of Quoy and Gaimard, e.g. by Pilsbry (1900: 5; see above under *Bothriembryon inflatus castaneus*). As Kendrick and Wilson have shown (1975: 310), the specimen on which Quoy and Gaimard based the name *castanea* was from the same locality as their *Helix melo*. See also *Bothriembryon inflatus castaneus* Pilsbry, 1900.

##### Current systematic position.

Bothriembryontidae, *Bothriembryon melo* (Quoy and Gaimard, 1832).

#### Bothriembryon inflatus conispira

Pilsbry, 1900

http://species-id.net/wiki/Bothriembryon_inflatus_conispira

Fig. 4D

Bothriembryon inflatus conispira Pilsbry 1900: 6, pl. 10 figs 15–17.

##### Type material.

ANSP 8453, lectotype.

##### Remarks.

This taxon was considered a synonym of *Bothriembryon bulla* (Menke, 1843) by B.J. Smith (1992: 102); it was grouped by Richardson (1995) with *Bothriembryon melo* (Quoy and Gaimard, 1832). We here tentatively follow the latter opinion; however, further study is needed to clarify the status of this taxon.

##### Current systematic position.

Bothriembryontidae, *Bothriembryon melo* (Quoy and Gaimard, 1832).

#### Bulimus dux

Pfeiffer, 1861

http://species-id.net/wiki/Bulimus_dux

Bulimus dux Pfeiffer 1861: 24; Breure and Ablett 2012: 15, figs 3A–B.

##### Type material.

NHMUK 19598, lectotype.

##### Current systematic position.

Bothriembryontidae, *Bothriembryon dux* (Pfeiffer, 1861).

#### Bothriembryon fuscus

Thiele, 1930

http://species-id.net/wiki/Bothriembryon_fuscus

Fig. 8A

Bothriembryon fuscus Thiele 1930: 588, fig. 68; Köhler 2007: 142, fig. 76.

##### Type material.

ZMB 67610, holotype.

##### Remarks.

As Köhler (2007) correctly stated, this species was described from a single (but immature) preserved specimen.

##### Current systematic position.

Bothriembryontidae, *Bothriembryon fuscus* Thiele, 1930.

#### Bulinus gunnii

Sowerby II in Strzelecki, 1845

http://species-id.net/wiki/Bulinus_gunnii

Fig. 3N

Bulinus gunnii Sowerby II in Strzelecki 1845: 298, pl. 9 fig. 5 [sic, 6].
Liparus gunnii Sowerby; Harris 1897: 3.
Bulimus [sic] *gunnii* (Sowerby); Kershaw 1987: 1, fig. 1.

##### Type material.

NHMUK PI OR 96907, holotype.

##### Remarks.

This taxon is probably of Pliocene age, found at Hobart, Tasmania in Yellow Limestone (Travertine). Kershaw (1987) copied Sowerby’s figure and discussed the relationship between this taxon and *Bothriembryon tasmanicus* (Pfeiffer, 1853). A photograph of the holotype of *Bothriembryon gunnii* is here presented for the first time since its original publication.

##### Current systematic position.

Bothriembryontidae, *Bothriembryon gunnii* (Sowerby II in Strzelecki, 1845).

#### Bothriembryon melo hartogensis

Kobelt, 1901

http://species-id.net/wiki/Bothriembryon_melo_hartogensis

Fig. 5D

Bothriembryon melo hartogensis Kobelt 1901 [1899–1902]: 770, pl. 112 figs 15–16; Neubert and Janssen 2004: 212, pl. 14 fig. 160.

##### Type material.

SMF 25909/1a, holotype.

##### Remarks.

We follow B.J. Smith (1992) for the systematic position of this taxon.

##### Current systematic position.

Bothriembryontidae, *Bothriembryon costulatus* (Lamarck, 1822).

#### Bulimus inflatus

Lamarck, 1822

http://species-id.net/wiki/Bulimus_inflatus

Fig. 5C

Bulimus inflatus Lamarck 1822: 122; Mermod 1951: 728, fig. 78.
Helix costulata ‘Férussac’ Lamarck 1822: 122 (in synonymy).

##### Type material.

MHNG INVE 51162, five syntypes.

##### Remarks.

Kendrick and Wilson (1975: 307–308) resolved the nomenclatural status of both Lamarckian taxa and have pointed out that the type material of *Bulimus inflatus* Lamarck is the same as *Helix costulata* Lamarck; see art. 11.6 jo. 50.7 jo. 72.4.3. ICZN. They considered Mermod’s treatment of this taxon as a lectotype designation (Kendrick and Wilson 1975: 317, pl. 3 figs 4a–4b). “Mermod was most likely acting as first reviser, but his intention was certainly not to designate a lectotype, even if his statement might be considered as an inadvertent lectotype designation.” (Y. Finet, pers. commun.). B.J. Smith (1992: 102–103) erroneously attributed this material to *Helix costulata* and considered the type material of *Bulimus inflatus* “presumably lost”.

##### Current systematic position.

Bothriembryontidae, *Bothriembryon costulatus* (Lamarck, 1822).

#### Bulimus kingii

J.E. Gray, 1825

http://species-id.net/wiki/Bulimus_kingii

Bulimus kingii J.E. Gray 1825: 414; Breure and Ablett 2012: 22, figs 2F–G, 2i

##### Type material.

NHMUK 195910, lectotype.

##### Current systematic position.

Bothriembryontidae, *Bothriembryon kingii* (J.E. Gray, 1825).

#### Bulimus (Liparus) leeuwinensis

E.A. Smith, 1894

http://species-id.net/wiki/Bulimus_leeuwinensis

Bulimus (Liparus) leeuwinensis E.A. Smith 1894: 94; Breure and Ablett 2012: 24, figs 3C–E.

##### Type material.

NHMUK 1891.11.21.128, lectotype.

##### Current systematic position.

Bothriembryontidae, *Bothriembryon leeuwinensis* (E.A. Smith, 1894).

#### Bothriembryon inflatus maculiferus

Pilsbry, 1900

http://species-id.net/wiki/Bothriembryon_inflatus_maculiferus

Fig. 6C

Bothriembryon inflatus maculiferus Pilsbry 1900: 5, pl. 1 figs 12–14; Baker 1963: 229.

##### Type material.

ANSP 8450a, lectotype.

##### Current systematic position.

Bothriembryontidae, *Bothriembryon rhodostomus* (J.E. Gray, 1834).

#### Bothriembryon martensi

Kobelt, 1901

http://species-id.net/wiki/Bothriembryon_martensi

Fig. 6H

Bothriembryon martensi Kobelt 1901 [1899–1902]: 764, pl. 112 figs 3–4; B.J. Smith 1992: 106; Köhler 2007: 143, fig. 77.

##### Type material.

ZMB 101818a, lectotype.

##### Remarks.

This taxon was placed in the synonymy of *Bothriembryon rhodostomus* (Gray, 1834) by B.J. Smith (1992: 106), apparently not based on examination of the type material. The figure shown by Köhler (2007; here reproduced) is a shell much larger (H = 44.5) than any of the taxa currently considered to be *Bothriembryon rhodostomus*, and the colouration is with darker tones. We tentatively follow here Smith’s judgement, but further studies should clarify the status of this taxon.

##### Current systematic position.

Bothriembryontidae, *Bothriembryon rhodostomus* (J.E. Gray, 1834).

#### Helix melo

Quoy & Gaimard, 1832

http://species-id.net/wiki/Helix_melo

Fig. 4A

Helix melo Quoy and Gaimard 1832: 109, pl. 9 figs 4–5.

##### Type material.

MNHN 24644, lectotype.

##### Current systematic position.

Bothriembryontidae, *Bothriembryon melo* (Quoy and Gaimard, 1832).

#### Bothriembryon onslowi minor

Pilsbry, 1900

http://species-id.net/wiki/Bothriembryon_onslowi_minor

Fig. 5E

Bothriembryon onslowi minor Pilsbry 1900: 12, pl. 3 figs 45–46; Kendrick and Wilson 1975: 309.

##### Type material.

ANSP 78504, two syntypes.

##### Remarks.

Kendrick and Wilson (1975) have argued that Iredale (1939) made a mistake when he raised this taxon to full specific status; they considered *minor* Pilsbry a junior subjective synonym of *Bothriembryon costulatus* (Lamarck). They also assumed that Pilsbry’s material was collected at Dirk Hartog Island; however, without positive evidence. Examining one of the syntypes the sculpture of the upper whorl shows a characteristic pattern of strong granules on the upper part of the whorls, and less strong, oblong granules on the lower part which are vanishing on the penultimate and ultimate whorl. This pattern may also be observed in *Bothriembryon mastersi* (Cox) and relatives. Since Pilsbry’s material did not have locality data and this sculpture is not observed in any of the species from Shark Bay, it cannot a priori be excluded that his specimens originated from a different place than the Shark Bay region. Therefore we only tentatively assign this taxon to *Bothriembryon costulatus*, awaiting further study to clarify the status of *Bothriembryon minor*.

##### Current systematic position.

Bothriembryontidae, *Bothriembryon ?costulatus* (Lamarck, 1822).

#### Bothriembryon kingii naturalistarum

Kobelt, 1901

http://species-id.net/wiki/Bothriembryon_kingii_naturalistarum

Fig. 7G

Bothriembryon kingii naturalistarum Kobelt 1901 [1899–1902]: 781, pl. 113 figs 22–23; Neubert and Janssen 2004: 219, pl. 14 fig. 161.

##### Type material.

SMF 25891a/1, syntype.

##### Current systematic position.

Bothriembryontidae, *Bothriembryon naturalistarum* Kobelt, 1901.

#### Bulimus physoides

Reeve, 1849

http://species-id.net/wiki/Bulimus_physoides

Bulimus physoides Reeve 1849 [1848–1850]: pl. 70 fig. 507; Breure and Ablett 2012: 31, figs 2H–I.

##### Type material.

NHMUK 1975224, three possible syntypes (Cuming coll.).

##### Remarks.

See the remarks in Breure and Ablett (2012) about the type status of the specimens and the relation with *Bulimus physodes* Menke, 1843. B.J. Smith (1992: 105) has synonymized Menke’s taxon with *Bothriembryon melo* (Quoy and Gaimard, 1832); E.A. Smith (1894: 95) mentioned several differences between the two taxa and gave Menke’s taxon full species status. We tentatively follow the opinion of B.J. Smith, but further studies are needed to solve the systematic arrangement of both taxa.

##### Current systematic position.

Bothriembryontidae, *Bothriembryon melo* (Quoy and Gaimard, 1832).

#### Bulimus rhodostomus

J.E. Gray, 1834

http://species-id.net/wiki/Bulimus_rhodostomus

Bulimus rhodostomus J.E. Gray 1834: 65; B.J. Smith 1992: 106; Breure and Ablett 2012: 35, figs 3F–G.

##### Type material.

NHMUK 1874.10.28.1, lectotype; 1975222, one paralectotype (ex Gray).

##### Current systematic position.

Bothriembryontidae, *Bothriembryon rhodostomus* (J.E. Gray, 1834).

#### Bulimus tasmanicus

Pfeiffer, 1853

http://species-id.net/wiki/Bulimus_tasmanicus

Bulimus tasmanicus Pfeiffer 1853: 260; Breure and Ablett 2012: 41, figs 3H–I.

##### Type material.

NHMUK 1981204, one syntype.

##### Current systematic position.

Bothriembryontidae, *Bothriembryon tasmanicus* (Pfeiffer, 1853).

#### Helix trilineata

Quoy & Gaimard, 1832

http://species-id.net/wiki/Helix_trilineata

Fig. 9A

Helix trilineata Quoy and Gaimard 1832: 107, pl. 9 figs 1–3.

##### Type material.

MNHN 24642, lectotype.

##### Current systematic position.

Bothriembryontidae, *Bothriembryon kingii* (Gray,  1825).

## Supplementary Material

XML Treatment for Bothriembryon balteolus

XML Treatment for Bothriembryon barretti

XML Treatment for Bothriembryon bradshawi

XML Treatment for Bothriembryon inflatus castaneus

XML Treatment for Bothriembryon consors

XML Treatment for Bothriembryon decresensis

XML Treatment for Bothriembryon distinctus

XML Treatment for Bothriembryon douglasi

XML Treatment for Bothriembryon esperantia

XML Treatment for Bothriembryon leeuwinensis eventus

XML Treatment for Bothriembryon franki

XML Treatment for Bothriembryon gardneri

XML Treatment for Bothriembryon glauerti

XML Treatment for Bothriembryon rhodostomus grantianus

XML Treatment for Bulimus (Liparus) gratwicki

XML Treatment for Bothriembryon rhodostomus hullianus

XML Treatment for Bothriembryon physoides humilis

XML Treatment for Bothriembryon barretti indictus

XML Treatment for Bothriembryon irvineanus

XML Treatment for Bothriembryon jacksoni

XML Treatment for Bothriembryon kendricki

XML Treatment for Bothriembryon kremnobates

XML Treatment for Bulimus mastersi

XML Treatment for Bothriembryon multispirus

XML Treatment for Bothriembryon notatus

XML Treatment for Bulimus onslowi

XML Treatment for Bulimus indutus pallidus

XML Treatment for Bothriembryon perditus

XML Treatment for Bothriembryon perobesus

XML Treatment for Bothriembryon rhodostomus perspectus

XML Treatment for Bothriembryon praecelsus

XML Treatment for Bothriembryon praecursor

XML Treatment for Bothriembryon revectus

XML Treatment for Bothriembryon richeanus

XML Treatment for Bothriembryon ridei

XML Treatment for Bothriembryon sedgwicki

XML Treatment for Bothriembryon serpentinus

XML Treatment for Bothriembryon kingii solidus

XML Treatment for Liparus spenceri

XML Treatment for Bothriembryon whitleyi

XML Treatment for Bothriembryon rhodostomus wrightianus

XML Treatment for Bulimus angasianus

XML Treatment for Bulimus baconi

XML Treatment for Bothriembryon gunni brachysoma

XML Treatment for Bulimus (Liparus) brazieri

XML Treatment for Helix melo castanea

XML Treatment for Bothriembryon inflatus conispira

XML Treatment for Bulimus dux

XML Treatment for Bothriembryon fuscus

XML Treatment for Bulinus gunnii

XML Treatment for Bothriembryon melo hartogensis

XML Treatment for Bulimus inflatus

XML Treatment for Bulimus kingii

XML Treatment for Bulimus (Liparus) leeuwinensis

XML Treatment for Bothriembryon inflatus maculiferus

XML Treatment for Bothriembryon martensi

XML Treatment for Helix melo

XML Treatment for Bothriembryon onslowi minor

XML Treatment for Bothriembryon kingii naturalistarum

XML Treatment for Bulimus physoides

XML Treatment for Bulimus rhodostomus

XML Treatment for Bulimus tasmanicus

XML Treatment for Helix trilineata

## Figures and Tables

**Figure 3.**
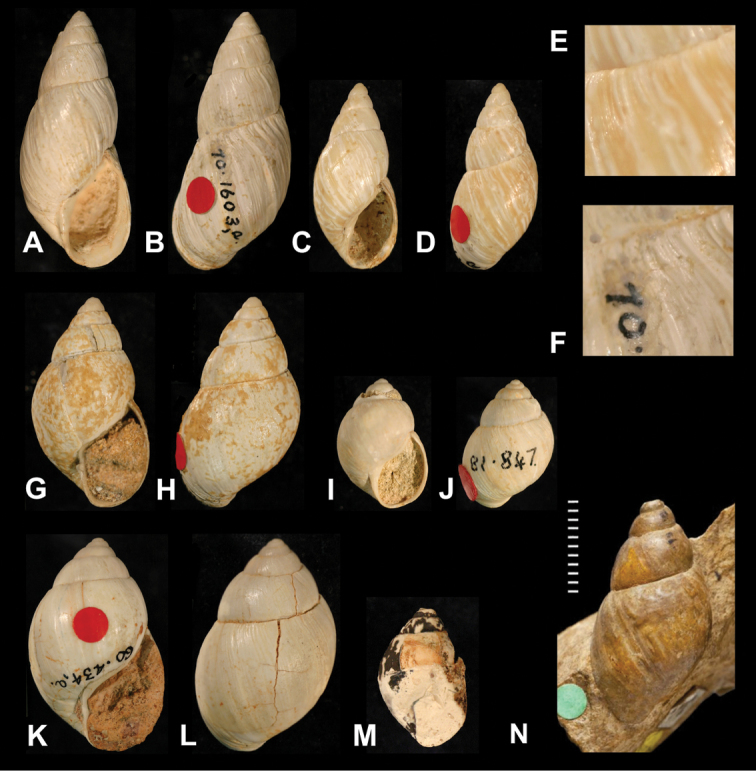
Fossil *Bothriembryon* species. **A, B, F** *B. gardneri* Kendrick, 1978, holotype, WAM 701603a (H = 43.5); **F** sculpture on dorsal side of last whorl **C–E** *B. consors* Kendrick, 1978, holotype, WAM 72421a (H = 30.0); **E** sculpture on dorsal side of last whorl **G–H** *B. douglasi* Kendrick, 1978, holotype, WAM 661036a (H = 34.2) **I–J** *B. kremnobates* Kendrick, 2005, holotype, WAM 81.847 (H = 21.5) **K–L** *B. ridei* Kendrick, 1978, holotype, WAM 60.434a (H = 36.6) **M** *B. praecursor* McMichael, 1968, holotype, CPC 6906 (H = 14.17) **N** *B. gunnii* (Sowerby II, 1845), holotype, NHMUK PI OR 96907 (H = 25.1).

**Figure 4.**
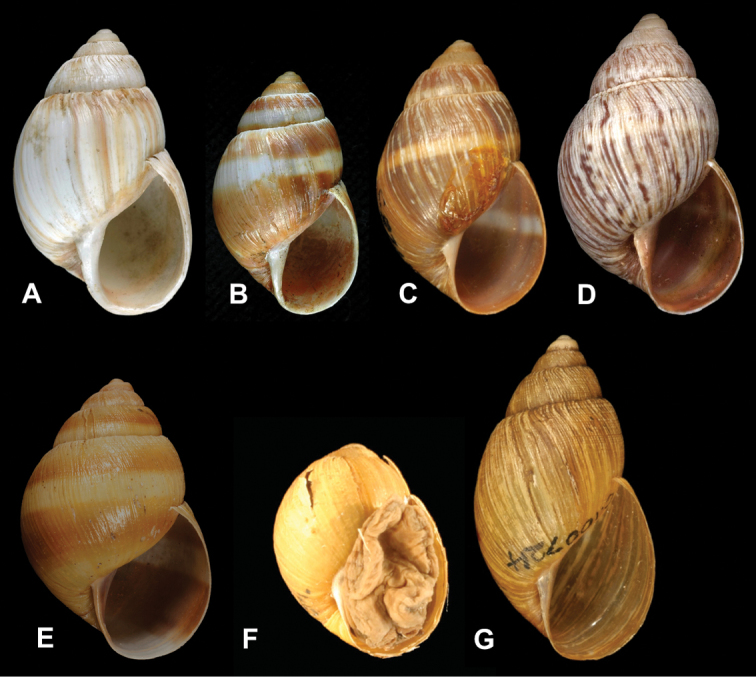
**A–D** *Bothriembryon melo* (Quoy and Gaimard, 1832) **A** Lectotype, MNHN 24644 (H = 24.7) **B** Paralectotype, *Helix melo castanea* Quoy and Gaimard, 1832, MNHN 24645 (H = 20.6) Photos: P. Maestrati **C** Syntype of *Bothriembryon inflatus castaneus* Pilsbry 1900, AM C87458 (H = 23.1) Photo: A. Miller **D** Lectotype of *B. inflatus conispira* Pilsbry, 1900, ANSP 8453 (H = 25.2) Photo: A. Lawless **E** *B. bulla* (Menke, 1843), holotype of *Bulimus baconi* Benson, 1854, UMZC 2397 (H = 23.6) Photo: R. Preece **F** *B. kendricki* Hill, Johnson & Merrifield, 1983, holotype, WAM S14552 (H = 16.2) **G** *B. serpentinus* Iredale, 1939, syntype, AM C100724 (H = 27.5).

**Figure 5.**
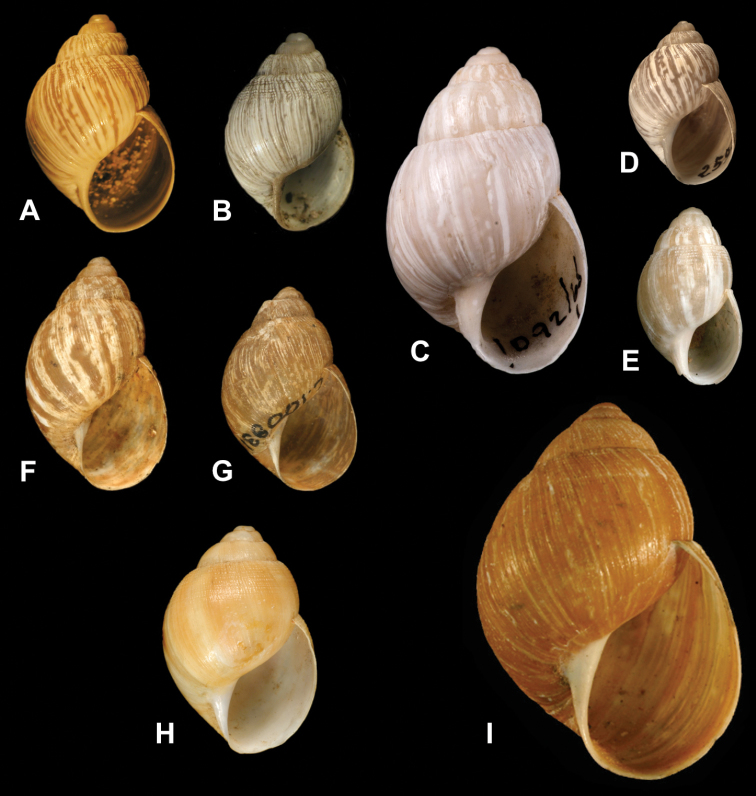
**A–B** *Bothriembryon mastersi* (Cox, 1867) **A** Possible syntype, SAMA D11341 (H = 17.4) **B** Holotype of *B. decresensis* Cotton, 1940, SAMA D13773 (H = 15.5) Photo: R. Hamilton-Bruce **C–E** *B. costulatus* (Lamarck, 1822) **C** Syntype of *Bulimus inflatus* Lamarck, 1822, MHNG INV 51162 (H = 26.0) Photo: Y. Finet **D** Holotype of *B. onslowi hartogensis* Kobelt, 1901, SMF 25909/1a (H = 12.9) Photo: E. Neubert **E** *S*yntype of *B. onslowi minor* Pilsbry, 1900, ANSP 78504 (H = 14.4) Photo: A. Lawless **F** *B. bradshawi* Iredale, 1939, syntype, AM C100749 (H = 19.0) **G** *B. sedgwicki* Iredale, 1939, syntype, AM C100831 (H = 15.9) **H** *B. onslowi* (Cox, 1864), syntype, AM C84882 (H = 21.7) **I** *B. praecelsus* Iredale, 1939, holotype, WAM S14468 (H = 27.1).

**Figure 6.**
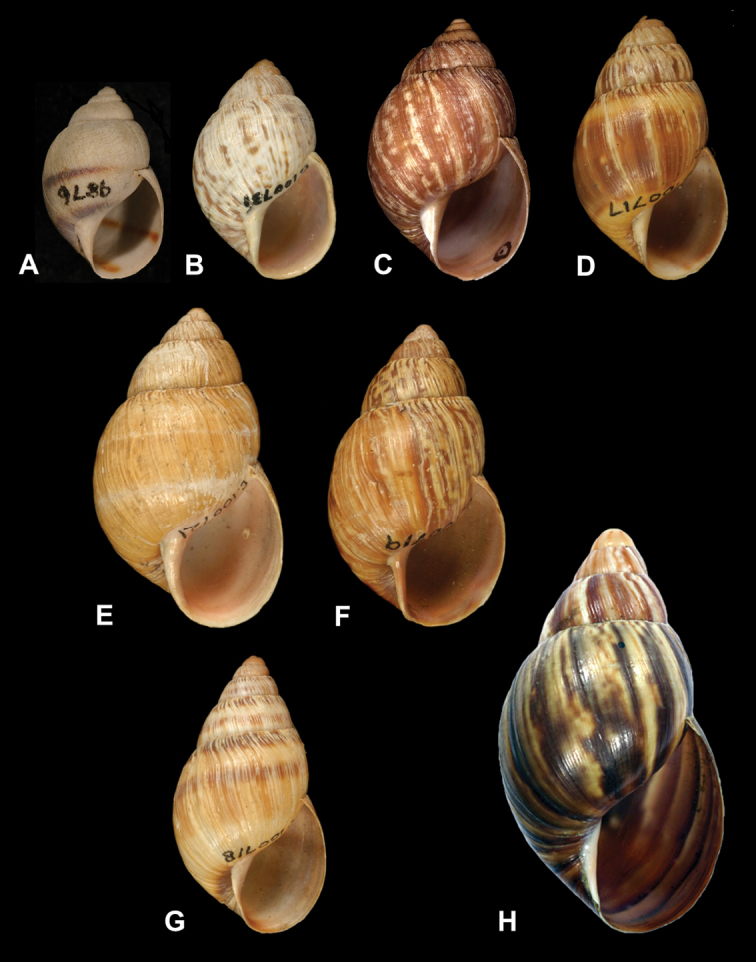
**A** *Bothriembryon balteolus* Iredale, 1939, lectotype, WAM S15154 (H = 21.5) **B** *B. esperantia* Iredale, 1939, syntype, AM C48681 (H = 23.9) **C–G** *B. rhodostomus* (Gray, 1834) **C** Lectotype of *B. inflatus maculiferus* Pilsbry, 1900, ANSP 8450 (H = 28.3) Photo: A. Lawless **D** Syntype of *B. rhodostomus wrightianus* Iredale, 1939, AM C100717 (H = 29.4) **E** Syntype of *B. rhodostomus grantianus* Iredale, 1939, AM C100721 (H = 36.5) **F** Syntype of *B. rhodostomus perspectus* Iredale, 1939, AM C100719 (H = 34.5) **G** Syntype of *B. rhodostomus hullianus* Iredale, 1939, AM C100718 (H = 31.7) **H** Lectotype of *B. martensi* Kobelt, 1901, ZMB 101818a (H = 44.5).

**Figure 8.**
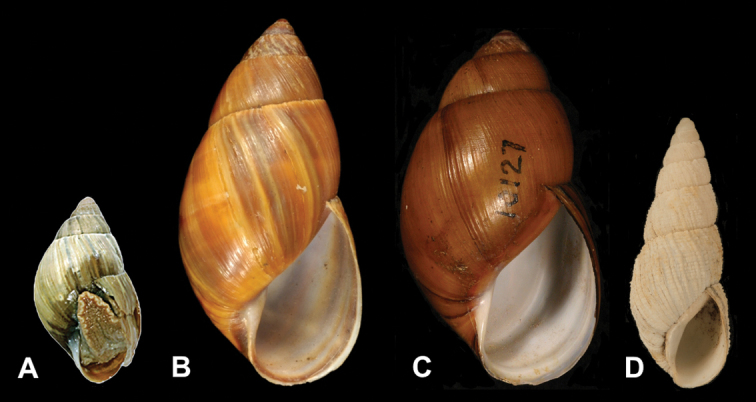
**A–B** *Bothriembryon fuscus* Thiele, 1930 **A** Holotype, ZMB 67610 (H = 20.0) Photo: F. Köhler **B** Syntype of *B. franki* Iredale, 1939, AM C100728 (H = 40.0) **C** *B. glauerti* Iredale, 1939, syntype, WAM S14466 (H = 36.7) **D** *Bulimus (Liparus) gratwicki* Cox, 1899, syntype, AM C127559 (H = 29.5).

**Figure 9.**
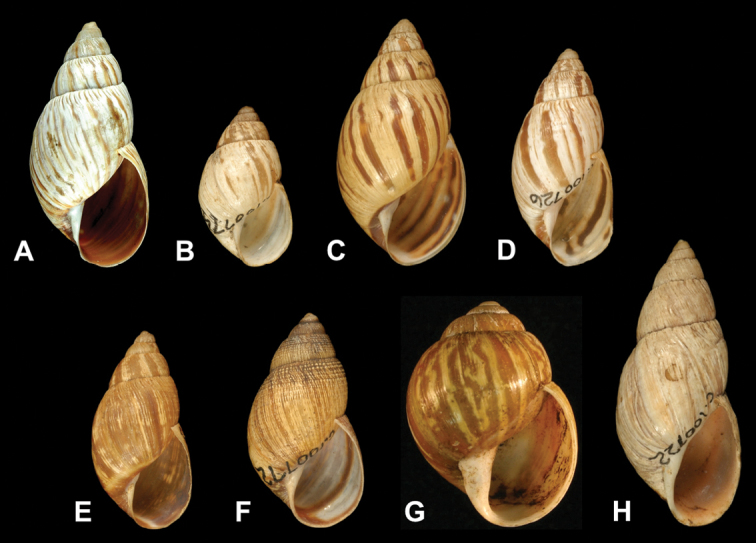
**A–E** Species complex of *Bothriembryon kingii* (J.E. Gray, 1825) **A** Lectotype of *Helix trilineata* Quoy and Gaimard, 1832, MNHN 24642 (H = 27.1) Photo: P. Maestrati **B** Lectotype of *B. physoides humilis* Pilsbry, 1900 AM C100774 (H = 17.2) **C** *B. jacksoni* Iredale, 1939, syntype, AM C100725 (H = 27.2) **D** *B. notatus* Iredale, 1939, syntype, AM C100726 (H = 23.6) **E** *B. revectus* Iredale, 1939, syntype, AM C100723 (H = 21.8) **F** *B. richeanus* Iredale, 1939, syntype, AM C100772 (H = 23.4) **G** *B. perobesus* Iredale, 1939, holotype, WAM S14467 (H = 24.2) **H** *B. sayi* (Pfeiffer, 1847), syntype of *B. kingii solidus* Pilsbry, 1900, AM C100722 (H = 31.8).

## References

[B1] Angas GF (1871) Descriptions of thirty-four new species of shells from Australia. Proceedings of the Zoological Society of London (1871): 13–21.

[B2] Baker HB (1963) Type land shells in the Academy of Natural Sciences of Philadelphia, II. Land Pulmonata, exclusive of North America north of Mexico. Proceedings of the Academy of Natural Sciences of Philadelphia 115: 191-259.

[B3] Benson WH (1854) Characters of a new European *Pupa*, and of a new Australian *Bulimus*. Annals and Magazine of Natural History (2) 13: 97–99. doi: 10.1080/03745485709495086

[B4] Breure ASH (1974) Caribbean land molluscs: Bulimulidae: I. *Bulimulus*. Studies on the Fauna of Curaçao and other Caribbean Islands 45: 1-80.

[B5] Breure ASH (1979) Systematics, phylogeny and zoogeography of Bulimulinae (Mollusca). Zoologische Verhandelingen Leiden 168: 1-215.

[B6] Breure ASH Ablett JD (2012) Annotated type catalogue of the Bothriembryontidae and Odontostomidae (Mollusca, Gastropoda, Orthalicoidea) in the Natural History Museum, London. ZooKeys 182: 1-70. doi: 10.3897/zookeys.182.2720 PMC3337044 22539914

[B7] Breure ASH Groenenberg DSJ Schilthuizen M (2010) New insights in the phylogenetic relations within the Orthalicoidea (Mollusca: Gastropoda), based on 28S sequence data. Basteria 74: 25-31.

[B8] Breure ASH Romero PE (in press) Support and surprises: molecular phylogeny of the land snail superfamily Orthalicoidea (Gastropoda, Stylommatophora) using a three-locus gene analysis with a divergence time analysis and ancestral area reconstruction.

[B9] Cotton BC (1940) The land shells of Kangaroo Island. The South Australian Naturalist 19: 40-43.

[B10] Cox JC (1864) Descriptions of twenty-six new species of Australian land-shells. Annals and Magazine of Natural History (3) 14: 185.

[B11] Cox JC (1867) Characters of four new species of Australian land-shells. Proceedings of the Zoological Society of London (1867): 39–40.

[B12] Cox JC (1899) Description of a new species of *Liparus* from West Australia. Proceedings of the Linnean Society of New South Wales 24: 435-436.

[B13] Crespin I (1974) Catalogue of additional type and figured specimens other than Protista in the Commonwealth Palaeontological Collection, Canberra. Bulletin Bureau of Mineral Resources, Geology and Geophysics 160: 1-161.

[B14] DEC (2010) Declared threatened fauna. http://www.dec.wa.gov.au/component/option,com_docman/task,doc_download/gid,4295/Itemid/ [accessed 25 October 2011]

[B15] Harris GF (1897) Catalogue of Tertiary Mollusca in the Department of Geology, British Museum (Natural History), I. The Australasian Tertiary Mollusca. Longmans and Co., London, xxvi + 407 pp.

[B16] Hill A Johnson MS Merrifield H (1983) An electrophoretic and morphological examination of *Bothriembryon kendricki* (Pulmonata: Bulimulidae), a new species previously considered conspecific with *B. bulla* (Menke). Australian Journal of Zoology 31: 227-242. doi: 10.1071/ZO9830227

[B17] Iredale T (1930) Notes on some desert snails. Victorian Naturalist (Melbourne) 47: 118-120.

[B18] Iredale T (1937) A basic list of the land Mollusca of Australia. The Australian Zoologist 8: 287–333.

[B19] Iredale T (1939) A review of the land Mollusca of Western Australia. Records of the Western Australian Museum and Art Gallery 2(1): 1–88. [Published 1.viii.1939; same text re-published 21.viii.1939 in Journal of the Royal Society of Western Australia 25:1–88 (read before the Society on 13.ix.1938, but publication delayed)].

[B20] Kendrick GW (1978) New species of fossil nonmarine molluscs from Western Australia and evidence of late Quarternary climatic change in the Shark Bay district. Journal of the Royal Society of Western Australia 60: 49-60.

[B21] Kendrick GW (2005) A new species of *Bothriembryon* (Mollusca: Gastropoda: Bulimulidae) from the Pliocene Roe Calcarenite, Eucla Basin, Western Australia. Records of the Western Australian Museum 22: 309-313.

[B22] Kendrick GW Wilson BR (1975) Nomenclatural notes on the land snail genus *Bothriembryon* Pilsbry, 1894 (Pulmonata: Bulimulidae), with redescriptions of the types and two other species. Records of the Western Australian Museum 3: 295-325.

[B23] Kershaw RC (1987) Type localities for six species of Tasmanian land molluscs (Pulmonata: Stylommatophora). Papers and Proceedings of the Royal Society of Tasmania 121: 57-68.

[B24] Kobelt W (1899–1902) Die Familie Buliminidae. In: Kuster HC, Kobelt W (eds.) Systematisches Conchylien-Cabinet von Martini und Chemnitz 1 (13, 2): 398–1051.

[B25] Köhler F (2007) Annotated type catalogue of the Bulimulidae (Pulmonata, Orthalicoidea, Bulimulidae) in the Museum für Naturkunde Berlin. Mitteilungen aus dem Museum für Naturkunde Berlin, Zoologische Reihe 83: 125-159.

[B26] Lamarck JBPA de (1822) Histoire naturelle des animaux sans vertèbres 6 (2). Verdières, Paris, 232 pp.

[B27] Macpherson JH (1951) Land Mollusca of the Russell Grimwade expedition. Memoirs of the National Museum Melbourne 17: 29-31.

[B28] McMichael DF (1968) Non-marine Mollusca from Tertiary rocks in Northern Australia. Bulletin Bureau of Mineral Resources, Geology and Geophysics 80: 135-159.

[B29] Neubert E Janssen R (2004) Die Typen und Typoide des Natur-Museums Senckenberg, 84: Mollusca: Gastropoda: Pulmonata: Orthalicoidea: Bulimulidae (2), Orthalicidae, Placostylidae. Archiv für Molluskenkunde 133: 193-297.

[B30] Pfeiffer L (1853) Monographia heliceorum viventium: sistens descriptiones systematicas et criticas omnium huius familiae generum et specierum hodie cognitarum, 3. Brockhaus, Lipsiae, 711 pp.

[B31] Pfeiffer L (1861) Description of fifty-seven new species of land-shells, from the collection of H. Cuming, Esq. Proceedings of the Zoological Society of London (1861): 20–29.

[B32] Pfeiffer L (1864) Description of ten new species of land-shells, from the collection of George French Angas, Esq. Proceedings of the Zoological Society of London (1863): 526–529.

[B33] Pilsbry HA (1900) Australasian Bulimulidae: *Bothriembryon*, *Placostylus*. Helicidae: *Amphidromus*. Manual of Conchology (2) 13: 1–253.

[B34] Quoy JCR Gaimard JP (1832) Mollusques, 1. In: Dumont d’Urville JSC, Voyage de la corvette l’Astrolabe, executé par l’ordre du Roi, pendant les années 1826–1827–1828–1829, Zoologie 2: 1–320. J. Tastu, Paris.

[B35] Richardson CL (1995) Bulimulidae: catalog of species. Tryonia 28: 1-458.

[B36] Shorthouse DP (2010) SimpleMappr, a web-enabled tool to produce publication-quality point maps. http://www.simplemappr.net [accessed 18 November 2011]

[B37] Smith BJ (1992) Non-marine Mollusca. In: Houston WWK (Ed) Zoological Catalogue of Australia, 8. Australian Government Publishing Service, Canberra, xii + 405 pp.

[B38] Smith EA (1894) On the land-shells of Western Australia. Proceedings of the Malacological Society of London 1: 84-99.

[B39] Strzelecki PE de (1845) Physical description of New South Wales and Van Diemen’s Land, accompanied by a geological map, sections and diagrams, and figures of the organic remains. Longman, Brown, Green, and Longmans, London, xix + 482 pp.

[B40] Tate R (1879) Descriptions of new species of South Australian pulmoniferous snails. Transactions and Proceedings and Report of the Philosophical Society of Adelaide, South Australia 1878–1879: 133–136.

[B41] Tate R (1894) Brief diagnoses of Mollusca from Central Australia. Transactions of the Royal Society of South Australia 18: 191-194.

[B42] Tate R (1896) Mollusca. In: Spencer WB (Ed) Report on the work of the Horn Scientific Expedition to Central Australia, 2. Zoology: 181–226. Melville, Mullen and Slade, London and Melbourne.

[B43] Wells FE (1977) Type specimens in the Department of Molluscs, Western Australian Museum. Records of the Western Australian Museum 6 (1): 33-61.

